# The Extracellular Matrix Proteins Pericardin and Lonely Heart Modulate the Directionality of Heart Contractions in Infected Mosquitoes

**DOI:** 10.1002/arch.70214

**Published:** 2026-09-03

**Authors:** Shabbir Ahmed, Puspa K. Shah, Julián F. Hillyer

**Affiliations:** ^1^ Department of Biological Sciences Vanderbilt University Nashville Tennessee USA

**Keywords:** *Anopheles gambiae*, contraction, dorsal vessel, extracellular matrix, heart, hemocyte

## Abstract

The cardiac extracellular matrix provides structural support to the primary circulatory organ of an insect: a dorsal vessel that is divided into an aorta in the thorax and a heart in the abdomen. In the fruit fly, *Drosophila melanogaster*, the extracellular matrix components, Pericardin and Lonely heart, are needed for proper heart contractility, as they mediate adhesion between cardiomyocytes, pericardial cells, hemocytes, and alary muscles. In the mosquito, *Anopheles gambiae*, Pericardin and Lonely heart are necessary for the infection‐induced migration of hemocytes to the heart. In these infected mosquitoes, hemocytes decrease the heart rate by releasing nitric oxide. Here, we demonstrate in mosquitoes that Pericardin is transcribed throughout the abdomen, but that Lonely heart is transcribed mainly in the heart. Moreover, we demonstrate that Pericardin and Lonely heart influence the infection‐induced disproportional decrease in the anterograde and retrograde heart rates. We also demonstrate that, in infected mosquitoes, Pericardin and Lonely heart alter the directionality of heart contractions, shifting to more contractions propagating anterograde. These findings further explain how the immune and circulatory systems of insects are functionally integrated by demonstrating that the cardiac extracellular matrix, in addition to driving hemocyte organization on the heart, modulates heart rhythmicity in infected mosquitoes.

## Research Highlights

- Pericardin is expressed throughout the abdomen, but Lonely heart is expressed mainly in the heart.
- Pericardin and Lonely heart influence the infection‐induced disproportional decrease in the heart rate.
- In infected mosquitoes, Pericardin and Lonely heart alter the directionality of heart contractions.

## Graphical abstract

Pericardin and Lonely heart, which are components of the cardiac extracellular matrix, influence the direction in which the heart of mosquitoes propels hemolymph (blood).

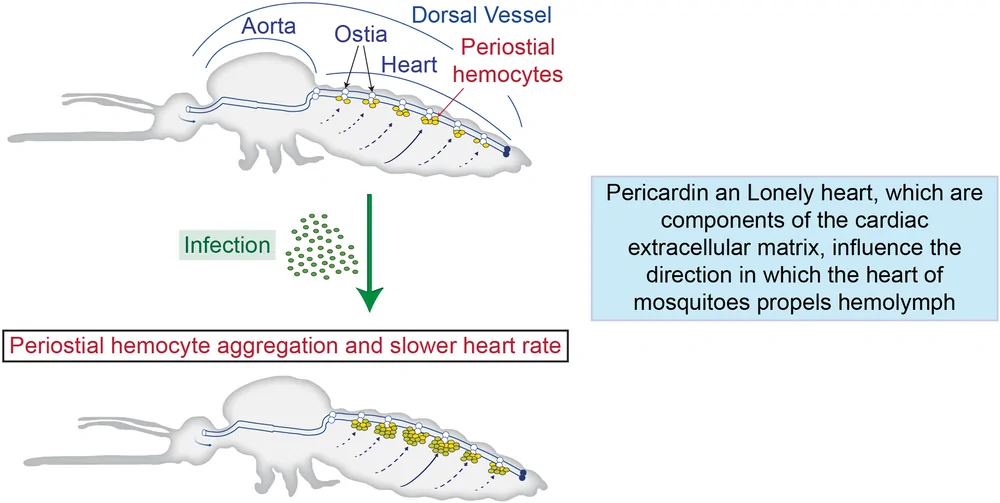

## 1 Introduction

The circulatory system of mosquitoes consists of hemolymph, an open body cavity known as the hemocoel, and a series of pumps, with the dorsal vessel being the most important (Hillyer and Pass 2020; Jones 1977). The dorsal vessel is a muscular tube that runs along the dorsal midline of the body and is divided into two segments: the aorta in the thorax and the heart in the abdomen (Barbosa Da Silva et al. 2019; Glenn et al. 2010; Sigle and Hillyer 2018b; Leódido et al. 2013). The heart alternates between contracting in anterograde and retrograde directions, propelling hemolymph toward the head and toward the posterior of the abdomen, respectively. During anterograde contractions, which in young mosquitoes occur two‐thirds of the time (Doran et al. 2017), hemolymph enters the dorsal vessel through paired valves, called ostia, in abdominal segments 2−7, and exits through an excurrent opening in the head (Glenn et al. 2010; Sigle and Hillyer 2018b). During retrograde contractions, which occur one‐third of the time, hemolymph enters through a pair of ostia at the thoraco‐abdominal junction and exits through an excurrent opening in the last abdominal segment. Once discharged into the hemocoel, hemolymph flows back toward the ostia to re‐enter the heart, completing the circulatory cycle. Although the insect heart contracts myogenically, neurohormones and neurotransmitters modulate both the speed and directionality of these contractions (Estévez‐Lao et al. 2013; Hillyer et al. 2015). Abiotic factors, such as nutritional status, and biotic factors, such as infection and aging, also impact circulatory physiology (Doran et al. 2017; Ellison et al. 2015; Estévez‐Lao et al. 2020).

The immune and circulatory systems of mosquitoes, and insects in general, are functionally integrated (King and Hillyer 2012; Yan and Hillyer 2020). Immune cells called hemocytes circulate with the hemolymph and also strategically attach to tissues throughout the body, becoming sessile (King and Hillyer 2013). During an infection, sessile hemocytes aggregate around the heart's ostia—locations called the periostial regions—where they release nitric oxide to lower the heart rate (Estévez‐Lao et al. 2020; King and Hillyer 2012; Sigle and Hillyer 2016; Sigle and Hillyer 2018a; Bergmann et al. 2024; Estévez‐Lao et al. 2025; Meier et al. 2025). The periostial regions are the only locations where hemocytes aggregate during an infection (King and Hillyer 2013), and they are ideal locations for hemocytes to aggregate because they place immune cells in the regions of the body with the highest hemolymph flow, where they are most likely to encounter pathogens circulating with the hemolymph (Sigle and Hillyer 2016).

The cardiac extracellular matrix (ECM) is an essential structural component of the heart. The cardiac ECM provides structural integrity, and it regulates cellular interactions during development (Hynes 2009; Rotstein and Paululat 2016). In the fruit fly, *Drosophila melanogaster*, the ECM proteins Pericardin and Lonely heart maintain cardiac structure by mediating adhesion between cardiomyocytes, pericardial cells, and alary muscles (Cevik et al. 2019; Drechsler et al. 2013; Reinhardt et al. 2023; Chartier et al. 2002). Loss of either gene leads to progressive heart failure. Pericardin and Lonely heart also influence how hemocytes organize on the heart of fruit flies, linking the cardiac ECM to immunity (Cevik et al. 2019). In adult mosquitoes, Pericardin and Lonely heart are positive regulators of the infection‐induced migration of hemocytes to the heart (Meier et al. 2024).

Here, we investigated the expression patterns of the genes that encode Pericardin (*Prc*) and Lonely heart (*Loh*) in the mosquito, *Anopheles gambiae*, and scrutinized whether manipulating their expression alters heart physiology. We demonstrate that *Prc* is expressed throughout the abdomen but that *Loh* is predominantly expressed on the heart, and that an infection either lowers their expression or leaves it unchanged. Finally, we demonstrate that disrupting Pericardin or Lonely heart does not affect heart rhythmicity in uninfected mosquitoes but affects the directionality of heart contractions in infected mosquitoes.

## 2 Materials and Methods

### 2.1 Mosquito Rearing

*A. gambiae* Giles sensu stricto (G3 strain; Diptera: Culicidae) were reared at 27°C, 75% relative humidity, and a 12 h:12 h light:dark photoperiod (Meier et al. 2024). Eggs were hatched in deionized water, and larvae were maintained on a diet of ground koi pellets and baker's yeast. Adults were maintained on 10% sucrose solution ad libitum. Experiments were initiated on 2‐day‐old adult females.

### 2.2 Synthesis of cDNA and Double‐Stranded Rna (dsRNA)

Total RNA was isolated from 10 whole adult females using TRIzol Reagent (Invitrogen, Carlsbad, CA, USA) and subsequently purified with the RNeasy Mini Kit (Qiagen, Valencia, CA, USA). Up to 5 µg of RNA was reverse‐transcribed using an Oligo(dT)_20_ primer and the SuperScript III First‐Strand Synthesis System for RT‐PCR (Invitrogen).

Gene‐specific primers containing T7 promoter sequences were designed to amplify *A. gambiae*'s Pericardin (*Prc*, AGAP007349) and Lonely heart (*Loh*, AGAP010003) (Supporting Information File S1). As a negative control, primers containing T7 promoter sequences were also designed to amplify beta‐lactamase (*Bla(Ap* ^*R*^ *)*) from *E. coli* BL21(DE3). Using *A. gambiae* cDNA or *E. coli* lysate as a template, amplicons were generated by PCR, resolved by agarose gel electrophoresis, excised, and purified using the QIAquick Gel Extraction Kit (Qiagen). Each purified fragment was reamplified by a second round of PCR to increase yield and used as a template for dsRNA synthesis with the MEGAscript T7 Kit (Applied Biosystems, Foster City, CA, USA). Synthesized dsRNA was ethanol‐precipitated, resuspended in phosphate‐buffered saline (PBS), and quantified spectrophotometrically.

### 2.3 RNA Interference and Bacterial Infection

For gene silencing, 300 ng of dsRNA was injected into the thoracic anepisternal cleft of 2‐day‐old females using a Nanoject III Programmable Nanoliter Injector (Drummond Scientific, Broomall, PA, USA). Mosquitoes were returned to 27°C, and 72 h later (at 5 days post‐emergence), a subset of mosquitoes was injured, and a subset of mosquitoes was infected by injecting bacteria.

Infections were performed with GFP‐expressing, tetracycline‐resistant *E. coli* (modified DH5‐α strain). Bacteria were cultured overnight in Luria–Bertani (LB) broth at 37°C, normalized to OD_600_ = 5.0, and 69 nL was injected intrathoracically into each mosquito. Heart physiology in naïve, injured (injected 69 nL of sterile LB broth), and infected mosquitoes was then measured at 6 days post‐emergence.

### 2.4 Quantification of RNAi Knockdown Efficiency

To measure RNAi‐based gene silencing efficiency, at 24 h after immune treatment (so, 4 days after dsRNA injection), the mosquitoes were bisected along the coronal plane, and the dorsal portion of the abdomen—which contains the heart, alary muscles, periostial hemocytes, pericardial cells, and other cells attached to the tergum—was collected in TRIzol reagent. We selected this time point because it was successfully used in our previous project that studied Pericardin and Lonely heart (Meier et al. 2024), and is consistent with methodologies used in mosquito research (Doran et al. 2017; Estévez‐Lao et al. 2013; Ramakrishnan and Hillyer 2022; Yan, Ramakrishnan, et al. 2022). A total of 20 dorsal abdomens were pooled per sample, and RNA was purified and cDNA synthesized as described above.

Quantitative PCR (qPCR) was conducted using gene‐specific primers (Supporting Information S1), Power SYBR Green Master Mix (Applied Biosystems), and a Bio‐Rad CFX Connect Real‐Time PCR System (Bio‐Rad, Hercules, CA, USA). Relative transcript abundance was calculated using the $2^{‐\Delta \Delta C_{T}}$ method (Livak and Schmittgen 2001), and the ribosomal gene, *RSP7*, was used as the reference (Estévez‐Lao et al. 2013). Fold‐change values were calculated relative to mosquitoes treated with the control dsRNA, ds*Bla(Ap* ^*R*^ *)*. Melting curve analysis confirmed that genomic DNA was absent from the cDNA preparations. Two independent biological trials were performed.

### 2.5 Analysis of Mosquito Heart Physiology

Cardiac physiology was analyzed by intravital imaging, as we have described (Andereck et al. 2010; Glenn et al. 2010; Meier et al. 2025). Mosquitoes were anesthetized for < 60 s on ice, positioned dorsal side up on a Sylgard 184 elastomer plate (Dow Corning Corp., Midland, MI), and restrained with 0.2 mm Minutien insect pins. Two pins were placed at a 45° angle against (not through) each side of the thoracic pronotal lobe, and one pin was placed through each wing.

After allowing mosquitoes to acclimate to room temperature for 5 min, intravital bright‐field videos of the dorsal abdomen were recorded for 65 s using a Nikon SMZ1500 stereomicroscope, a Hamamatsu ORCA‐Flash 2.8 CMOS camera, and Nikon NIS‐Elements Advanced Research software. The first 60 s of each video were manually analyzed in NIS‐Elements to measure the following cardiac parameters: (i) total contraction rate (regardless of contraction direction), (ii) anterograde and retrograde contraction rates, (iii) proportion of contractions that propagate anterograde versus retrograde, (iv) proportion of time the heart spends contracting anterograde versus retrograde, and (v) frequency of heartbeat directional reversals. For each dsRNA treatment, heart physiology was assessed in naïve, injured, and *E. coli*‐infected mosquitoes. Three biological replicates were performed per treatment, with 22–33 mosquitoes per treatment group.

### 2.6 Sampling, Data Availability, and Statistical Analysis

All datasets are provided in Supporting Information S1. Statistical analyses were conducted in GraphPad Prism v10.6.1 (GraphPad Software LLC, Boston, MA, USA). Heart physiology data were analyzed using two‐way ANOVA. This test yields three distinct *p*‐values, which in this study address (1) whether the type of dsRNA injected affects the results (ds*Bla(Ap* ^*R*^ *)*, ds*Prc*, ds*Loh*), (2) whether immune treatment affects the results (naïve, injured, infected), and (3) whether the type of dsRNA alters the effect of immune treatment and vice versa (interaction). Data in the graphs are presented as mean ± standard error of the mean (SEM), and statistical significance was accepted at *p* < 0.05.

## 3 Results

### 3.1 Pericardin Is Expressed Throughout the Abdomen While Lonely Heart Is Expressed in the Heart

In an earlier study, we generated an RNAseq data set that compared gene expression in response to *E. coli* and *S. aureus* infection in the heart and associated tissues, the circulating hemocytes, and the entire abdomen (Yan, Sigle, et al. 2022). In that dataset, the number of uniquely mapped reads per sample averaged 23,698,077 (range: 18,487,153–31,487,002), and differential expression was calculated based on reads per kilobase per million by DESeq. 2. Thus, we began the present study by mining this RNAseq dataset (SRA data PRJNA730047 in NCBI) to determine where *Prc* and *Loh* are expressed, and how they are regulated in response to infection (Table 1).

**Table 1:** Pericardin and Lonely heart gene expression in the heart and associated tissues, the circulating hemocytes, and the abdomen of naïve, injured and infected mosquitoes.^a^

Gene ID^b^	Gene	Tissue	Gene‐specific reads	*E. coli* versus naive		*S. aureus* versus naive	
				Log2 fold change	Adjusted *p*	Log2 fold change	Adjusted *p*
AGAP007349	*Prc*	Heart and associated tissues	13,065	−0.62	0.103	−1.02	0.004
AGAP007349	*Prc*	Circulating hemocytes	23,380	−2.10	3E‐10	−1.83	4E‐08
AGAP007349	*Prc*	Abdomen	8550	−0.60	0.199	−1.09	0.006
AGAP010003	*Loh*	Heart and associated tissues	20,107	0.05	0.930	−0.51	0.286
AGAP010003	*Loh*	Circulating hemocytes	71	0.09	0.942	−0.26	0.793
AGAP010003	*Loh*	Abdomen	7037	−0.92	0.070	−0.59	0.404

^a^ Data mined from SRA data PRJNA730047 in NCBI (https://www.ncbi.nlm.nih.gov).

^b^ Gene IDs are from the AgamP4 assembly of VectorBase (www.vectorbase.com).

Pericardin was expressed in all three tissues that were assayed. Adding the gene‐specific reads across all samples, there were 13065, 23380, and 8550 reads of *Prc* in the heart and associated tissues, circulating hemocytes, and abdomen (the abdomen contains the heart and associated tissues), respectively. Following infection with *E. coli*, *Prc* expression was reduced by ~35% in the heart and abdomen, and by 78% in the circulating hemocytes. Following infection with *S. aureus*, *Prc* expression decreased by ~50% in the heart and abdomen, and by 72% in the circulating hemocytes.

Lonely heart was expressed primarily in the heart and associated tissues. Across all samples, there were 20,107, 71, and 7037 reads of *Loh* in the heart and associated tissues, circulating hemocytes, and abdomen, respectively. We infer that most of the reads in the abdomen originate from the heart and associated tissues. Following infection with *E. coli*, *Loh* expression was unchanged in the heart and circulating hemocytes, but reduced by 47% in the abdomen. Following infection with *S. aureus*, *Loh* expression decreased by ~30% in the heart and abdomen, and by 16% in the circulating hemocytes. Overall, Pericardin is expressed throughout the abdomen, while Lonely heart is expressed in the heart. Moreover, an infection does not affect Lonely heart expression, and either does not affect or reduces the expression of Pericardin.

### 3.2 Prc and Loh Are Efficiently Silenced by RNAi

**Figure 1:**
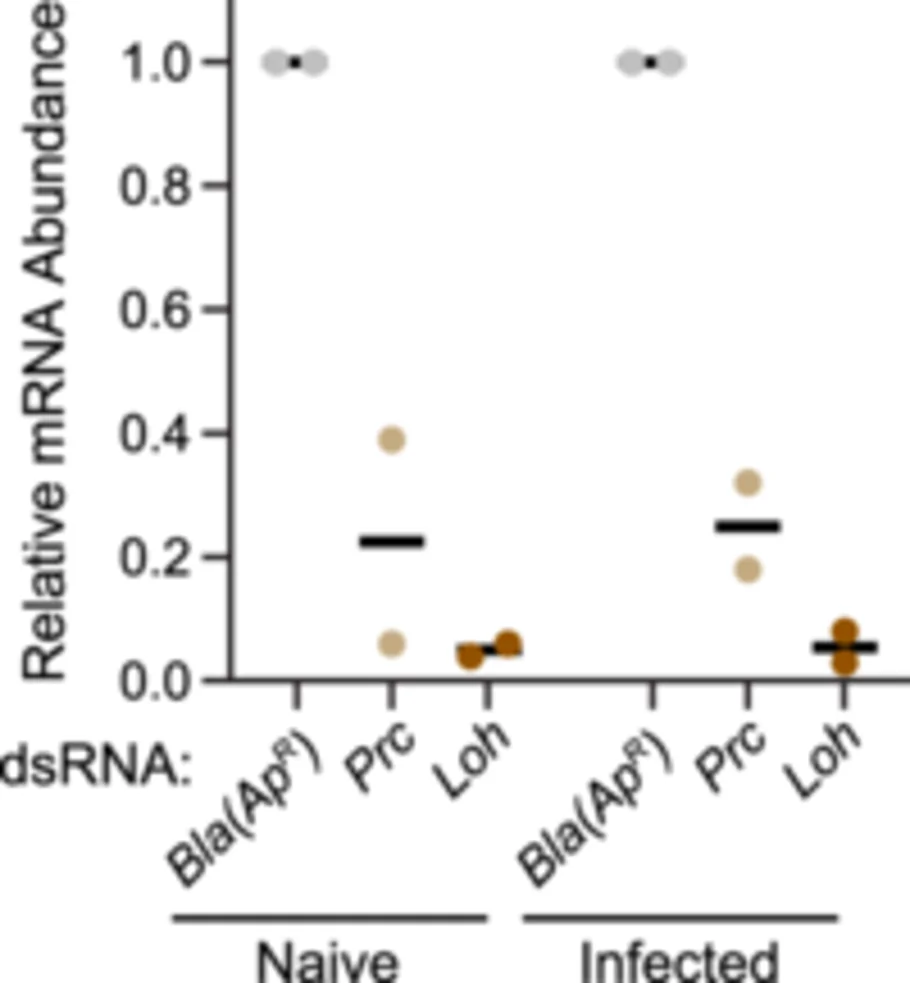
Pericardin and Lonely heart gene silencing efficiency by RNAi. Circles denote the individual data points and the horizontal lines the averages.

To measure the efficiency of *Prc* and *Loh* silencing by RNAi, mosquitoes were injected with gene‐specific or control dsRNA, and mRNA abundance was quantified in the dorsal abdomen—which contains the heart, pericardial cells, and periostial hemocytes—by qPCR at 4 days after treatment (Figure 1). Relative to ds*Bla(Ap* ^*R*^ *)*‐treated mosquitoes, treatment with ds*Prc* reduced *Prc* mRNA abundance by 64% in uninfected mosquitoes, and by 88% in mosquitoes that had been infected for 24 h. Likewise, relative to ds*Bla(Ap* ^*R*^ *)*‐treated mosquitoes, treatment with ds*Loh* reduced *Loh* mRNA abundance by 95% in uninfected mosquitoes, and by 94% in mosquitoes that had been infected for 24 h. These results demonstrate that dsRNA treatment effectively reduces *Prc* and *Loh* mRNA levels. The silencing efficiency observed in this study is similar to the silencing efficiency we observed in our earlier study, where mRNA abundance of *Prc* was reduced 75% in both naive and infected mosquitoes, and mRNA abundance of *Loh* was reduced 60% and 72% in naive and infected mosquitoes, respectively (Meier et al. 2024).

### 3.3 Perturbing Prc or Loh Influences How an Infection Disproportionally Decreases the Anterograde and Retrograde Heart Rates

**Figure 2:**
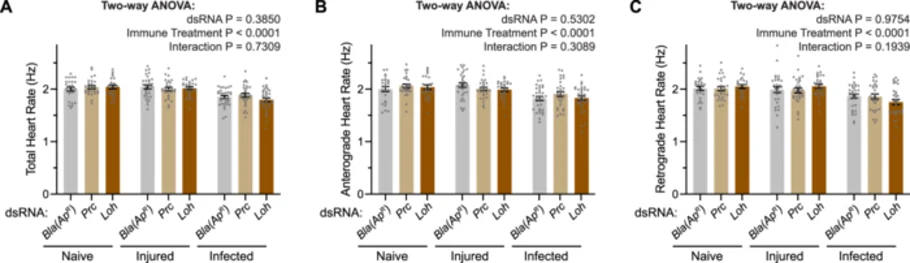
Effect of RNAi‐based silencing of Pericardin and Lonely heart on the heart rate of naïve, injured and infected mosquitoes. Parameters measured were the total heart rate (A; disregards contraction direction), the anterograde heart rate (B), and the retrograde heart rate (C). Column heights and whiskers mark the means ± SEM. Circles denote the individual data points.

Prc and Loh influence the aggregation of hemocytes on the heart of mosquitoes (Meier et al. 2024), and their disruption affects heart rhythmicity in *Drosophila* (Cevik et al. 2019; Drechsler et al. 2013; Reinhardt et al. 2023). Therefore, we tested whether silencing these genes affects circulatory physiology in *A. gambiae*. The heart rate of naïve mosquitoes was similar regardless of dsRNA treatment: the heart of mosquitoes treated with ds*Bla(Ap* ^*R*^ *)*, ds*Prc* or ds*Loh* contracted at an average of 2.00, 2.04 and 2.04 Hz, respectively (Figure 2A). Injury had a small effect on the heart rate, but for all dsRNAs, infection decreased the heart rate (Figure 2A). Relative to injured mosquitoes (infection requires that mosquitoes be injured), the heart rate of infected mosquitoes treated with ds*Bla(Ap* ^*R*^ *)*, ds*Prc* or ds*Loh* contracted 9%, 6%, and 11% slower. Overall, neither silencing *Prc* nor *Loh* affected the heart rate (two‐way ANOVA for dsRNA *p* = 0.7309), but infection decreased the heart rate (two‐way ANOVA for immune treatment *p* < 0.0001). Moreover, dsRNA treatment did not interact with immune treatment to modify the heart rate (two‐way ANOVA for interaction *p* = 0.3850), which indicates that the effect of infection is similar for all dsRNA treatments.

The mosquito heart contracts in both anterograde and retrograde directions (Andereck et al. 2010; Glenn et al. 2010), so we separated the data by contraction directionality and conducted another analysis (Figure 2B,C). The heart rate of naïve mosquitoes treated with ds*Bla(Ap* ^*R*^ *)*, ds*Prc* or ds*Loh* was similar when contracting in the anterograde direction (2.00, 2.05, and 2.03 Hz, respectively) as well as when it was contracting in the retrograde direction (2.02, 2.01, and 2.05 Hz, respectively). Moreover, regardless of dsRNA treatment, an infection reduced both the anterograde and retrograde contraction rates. However, we observed a subtle difference on how gene silencing impacted the effect of infection. Prior studies have shown that an infection causes a more pronounced reduction in the contraction rate when the heart contracts anterograde relative to when it contracts retrograde (Estévez‐Lao et al. 2020; Meier et al. 2025). This was indeed observed when mosquitoes were treated with ds*Bla(Ap* ^*R*^ *)*: there was a 12% reduction in the anterograde heart rate versus a 6% reduction in the retrograde heart rate. But this was not observed when mosquitoes were treated with ds*Prc* or ds*Loh*; compared to the injured group, when *Prc* was silenced, the infection‐induced reduction in the anterograde (5%) and retrograde (6%) heart rates were similar, and when *Loh* was silenced, the decrease in the heart rate was smaller when contracting anterograde (8%) than when contracting retrograde (15%). Therefore, silencing Pericardin and Lonely heart influences how an infection disproportionally decreases the anterograde and retrograde heart rates.

### 3.4 Perturbing Prc or Loh Shifts the Heart to Contracting More Anterograde in Infected Mosquitoes

About two thirds of heart contractions in a 5‐day‐old mosquito propel hemolymph in the anterograde direction, and about one third propel hemolymph in the retrograde direction (Andereck et al. 2010; Doran et al. 2017; Glenn et al. 2010). Because the anterograde and retrograde contraction rates are similar, these proportions also apply to the percentage of time the heart contracts anterograde versus retrograde.

**Figure 3:**
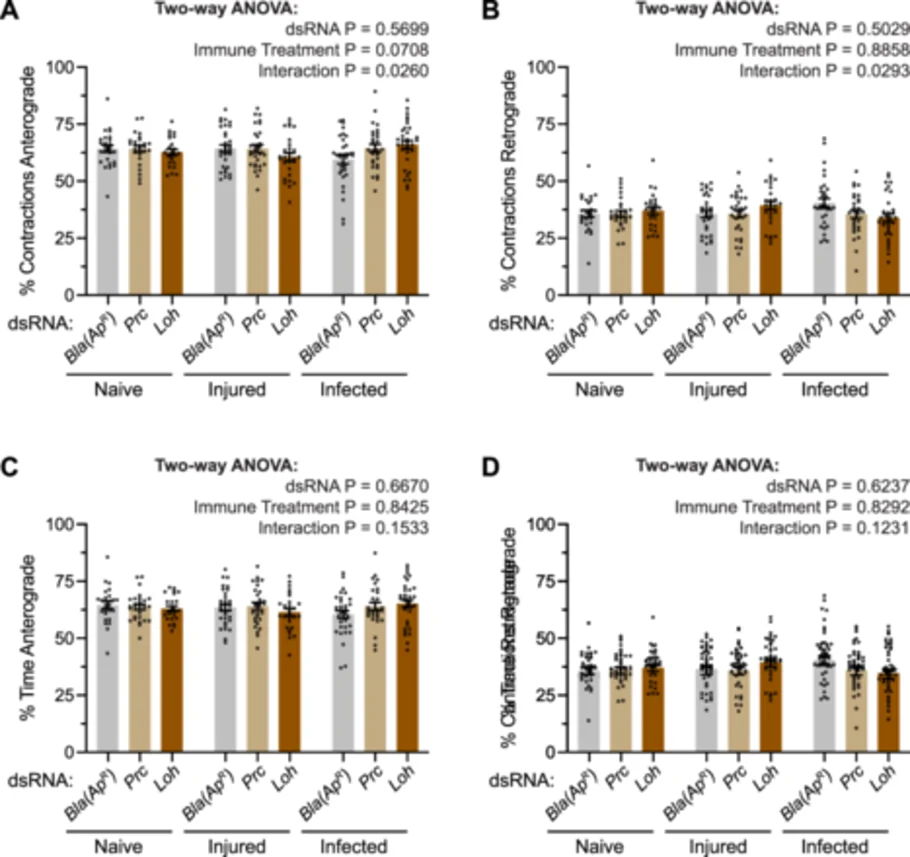
Effect of RNAi‐based silencing of Pericardin and Lonely heart on the directionality of heart contractions in naïve, injured and infected mosquitoes. Parameters measured were the percentage of contractions that propagate anterograde (A) and retrograde (B), and the percentage of the time the heart contracts anterograde (C) and retrograde (D). Column heights and whiskers mark the means ± SEM. Circles denote the individual data points.

In naïve mosquitoes, dsRNA treatment did not affect the proportion of contractions propagating in a given direction or the proportion of time that the heart contracted anterograde versus retrograde (Figure 3). In mosquitoes treated with ds*Bla(Ap* ^*R*^ *)*, ds*Prc* or ds*Loh*, 64%, 64%, and 63% of contractions propagated anterograde, respectively. Likewise, the heart spent 65%, 64%, and 63% of the time contracting anterograde. The remaining contractions and the remaining time contracted retrograde.

In infected mosquitoes, treatment with ds*Bla(Ap* ^*R*^ *)* reduced the percentage of contractions propagating anterograde (7% fewer contractions) as well as the percentage of time the heart contracted anterograde (5% less time). Infection did not alter these parameters in mosquitoes treated with ds*Prc* (0% change), but treatment with ds*Loh* increased the percentage of contractions propagating anterograde (9% more contractions) as well as the percentage of time the heart contracted anterograde (6% more time). Altogether, this indicates that heart contraction directionality is altered by the specific dsRNA that was administered (Figure 3; two‐way ANOVA for interaction: percentage contractions anterograde *p* = 0.0260; percentage time anterograde *p* = 0.1533). More specifically, in infected mosquitoes, silencing *Loh*, and to a lesser extend *Prc*, shifts the heart so that it contracts more in the anterograde direction.

### 3.5 Infection Increases the Frequency of Heartbeat Directional Reversals, but Perturbing Prc or Loh Does Not Have a Meaningful Effect

**Figure 4:**
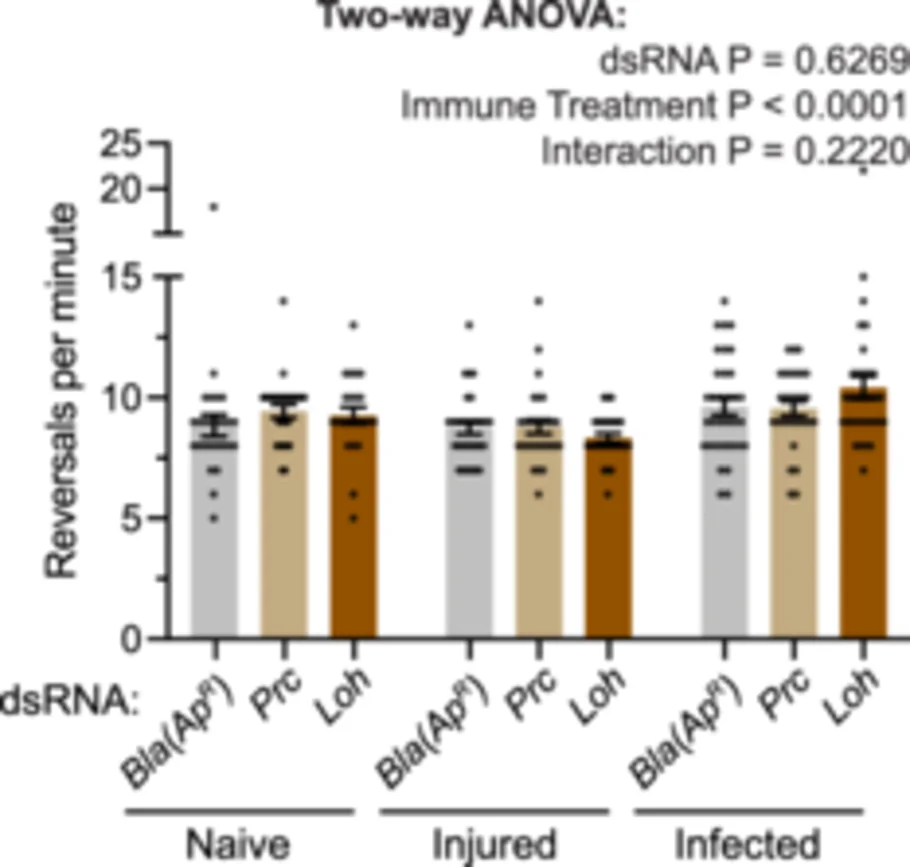
Effect of RNAi‐based silencing of Pericardin and Lonely heart on the frequency of heartbeat directional reversals in naïve, injured and infected mosquitoes. Column heights and whiskers mark the means ± SEM. Circles denote the individual data points.

Naïve and injured mosquitoes treated with ds*Bla(Ap* ^*R*^ *)*, ds*Prc* or ds*Loh* had a similar frequency of heartbeat directional reversals: 9 every minute (Figure 4). In ds*Bla(Ap* ^*R*^ *)* and ds*Prc* treated mosquitoes, an infection increased the frequency of heartbeat directional reversals by 9%, but in ds*Loh* treated mosquitoes, this increase was 23%. Of all cardiac parameters, the frequency of heartbeat directional reversals tends to be most variable (Andereck et al. 2010; Estévez‐Lao et al. 2013; Glenn et al. 2010). The infection induced increase in the frequency of heartbeat directional reversals (two‐way ANOVA for infection *p* < 0.0001) follows a pattern that we have observed after infection with *E. coli* (Estévez‐Lao et al. 2020; Meier et al. 2025), but the type of dsRNA treatment (*p* = 0.6269), or the interaction between dsRNA treatment and infection status (*p* = 0.2220), did not have a meaningful effect on this cardiac parameter.

## 4 Discussion

In fruit flies, the ECM components Pericardin (Prc) and Lonely heart (Loh) mediate adhesion between cardiomyocytes, pericardial cells and alary muscles, and they affect how hemocytes assemble on the surface of the heart (Cevik et al. 2019; Chartier et al. 2002; Drechsler et al. 2013; Reinhardt et al. 2023). Because of their essential roles in maintaining cardiac structural integrity, loss of Pericardin (Prc) or Lonely heart (Loh) causes cardiac dysfunction in fruit flies (Drechsler et al. 2013). In mosquitoes, the functions of Pericardin and Lonely heart are less understood, but they share significant sequence identity with their *Drosophila* homologs and are necessary for the infection‐induced migration of hemocytes to the heart (Meier et al. 2024). Here, we demonstrate in mosquitoes that Pericardin is transcribed throughout the abdomen, but that Lonely heart is transcribed mainly in the heart. We also demonstrate that, in infected mosquitoes, Pericardin and Lonely heart influence the disproportional decrease in the anterograde and retrograde heart rates, and shift the heart to contracting more in the anterograde direction.

In *Drosophila*, Pericardin is expressed in pericardial cells during heart development, and afterwards it is produced in the fat body (Chartier et al. 2002; Drechsler et al. 2013). Lonely heart is produced by cardiomyocytes, where it functions as the receptor that recruits Pericardin to the heart (Drechsler et al. 2013). Gene expression data suggest that this is also the case in mosquitoes. Specifically, *Prc* mRNA is abundant in the heart and associated tissues, the circulating hemocytes, and the abdomen. *Loh* mRNA, however, is most abundant in the heart and associated tissues. It is also present in the abdomen (which contains the heart) but not in the circulating hemocytes. Interestingly, an infection downregulates the expression of *Prc* but not *Loh*, a finding whose functional significance could not be confidently deciphered in this study. However, if *Prc* expression is reduced, then the cardiac ECM would likely be modified even if *Loh* expression is unchanged because less Prc would be captured and incorporated into the heart.

In insects, the cardiac ECM forms a scaffold that anchors the heart to the surrounding tissues, including the alary muscles and epidermis (Rotstein and Paululat 2016; Volk et al. 2014). This ECM maintains the physical alignment of the cardiac apparatus while at the same time providing a structural framework for other associated cells, such as hemocytes and pericardial cells. Disruption of Pericardin and Lonely heart, which are key components of the ECM, causes the progressive detachment of pericardial cells, a loss of cardiac elasticity, and the eventual collapse of the heart (Cevik et al. 2019; Drechsler et al. 2013). Similarly, mutants lacking other ECM elements—including LamininB1, LamininB2, and Collagen IV—exhibit structural disorganization and cardiac failure, underscoring the importance of ECM integrity for maintaining heart function (Hollfelder et al. 2014; Wolfstetter and Holz 2012). Collectively, these studies demonstrate that the cardiac ECM is essential for maintaining cardiac organization, and importantly, heart rhythmicity.

Heart dysfunction in fruit flies that lack Pericardin and Lonely heart is characterized by changes in the wave‐like contraction of the heart, with periods of systole and diastole not clearly discernible (Drechsler et al. 2013). The result is impaired intracardiac anterograde hemolymph flow (retrograde flow was not visualized in that study). These experiments relied on Pericardin and Lonely heart mutants, an approach we did not pursue in mosquitoes. Instead, we used RNAi to reduce (not eliminate) mRNA level of *Prc* or *Loh* in fully developed adults. We found that silencing these genes modulates cardiac physiology but only in infected mosquitoes. Specifically, silencing Pericardin and Lonely heart influences how an infection disproportionally decreases the anterograde and retrograde heart rates, and the directionality of heart contractions. In mosquitoes with normal expression of *Prc* and *Loh*, an infection causes a disproportionate decrease in the anterograde heart rate relative to the retrograde heart rate (Estévez‐Lao et al. 2020; Meier et al. 2025). This effect disappears when *Prc* is silenced, and is reversed when *Loh* is silenced. Moreover, in infected mosquitoes, silencing *Prc* and *Loh* increases the proportion of contractions that propagate anterograde.

The mechanisms by which Pericardin and Lonely heart modulate the heart in a contraction‐direction‐specific manner could be due to their effect on the periostial hemocytes that flank the heart. Silencing *Prc* and *Loh* decreases the infection‐induced migration of hemocytes to the heart of mosquitoes (Meier et al. 2024). Fewer periostial hemocytes means that less nitric oxide is produced on the heart (Estévez‐Lao et al. 2020; Meier et al. 2025), and nitric oxide reduces the heart rate of insects (Broderick et al. 2006; Da Silva et al. 2012; Estévez‐Lao et al. 2020). The effect of infection (and nitric oxide) on the heart rate is more pronounced when the heart contracts anterograde (Estévez‐Lao et al. 2020; Meier et al. 2025), and this directional phenotype is abrogated when Pericardin or Lonely heart are perturbed. Together, these results suggest that Pericardin and Lonely heart function to maintain the structural organization of the cardiac ECM, and that the disruption that occurs after gene silencing disrupts the immunological organization of the heart, thereby altering circulatory physiology.

The findings in this study illustrate that Pericardin and Lonely heart modulate circulatory physiology in infected mosquitoes but not in uninfected mosquitoes. This is different from the findings in *Drosophila*, where heart rhythmicity was affected in uninfected fruit flies (infected fruit flies were not assayed). These differences may be explained by the difference in the experimental approach of our study relative to how experiments were conducted in fruit flies. Our study used RNAi in adult mosquitoes. Thus, our experiments were initiated in insects with a fully developed heart and ECM, and the injection of dsRNA altered how new components were added to a fully formed ECM. The experiments in fruit flies used genetic mutants of *Prc* and *Loh*, indicating that their disruption occurred throughout development and adulthood, and that the level of disruption was more severe (Drechsler et al. 2013). Therefore, based on our data, we can conclude that Pericardin and Lonely heart impact circulatory physiology in infected mosquitoes, but we cannot discount the possibility that Pericardin and Lonely heart also influence circulatory physiology in uninfected mosquitoes.

The present study exclusively focused on the role that Pericardin and Lonely heart play in cardiac physiology, but alterations in heart function can influence other aspects of insect biology and fitness. In insects, the circulatory system contributes not only to immune responses but also to nutrient transport, homeostasis, thermoregulation, gas exchange, and locomotion (Hillyer and Pass 2020). In *Drosophila*, Pericardin and Lonely heart maintain cardiac integrity and function, and manipulating the cardiac ECM can improve lifespan (Rotstein et al. 2018; Sessions et al. 2017; Cevik et al. 2019; Reinhardt et al. 2023). Thus, it is possible that Pericardin and Lonely Heart may affect physiological processes linked to flight performance, activity, reproduction, or survival in mosquitoes. No obvious defects in mosquito viability or behavior were observed during this study, but those phenotypes were not our focus. Future work should investigate the broader effects of disrupting the cardiac ECM of mosquitoes.

In conclusion, the ECM proteins Pericardin and Lonely heart modulate the directionality of heart contractions in infected mosquitoes. The IMD pathway as well as transglutaminases also affect both the immunological organization of the heart and heart physiology (Estévez‐Lao et al. 2025; Ramakrishnan and Hillyer 2022; Yan, Ramakrishnan, et al. 2022; Yan, Sigle, et al. 2022). Here, we expand our knowledge of how the immune and circulatory systems of insects are functionally integrated by demonstrating that structural components of the cardiac ECM, in addition to driving how hemocytes are recruited to the heart in response to infection (Meier et al. 2024), modulate heart rhythmicity in infected mosquitoes.

## Author Contributions

**Shabbir Ahmed:** writing – original draft, conceptualization, investigation, methodology, validation, visualization, writing – review and editing, formal analysis, data curation, project administration. **Puspa K. Shah:** writing – original draft, investigation, methodology, validation, writing – review and editing, formal analysis, data curation, visualization. **Julián F. Hillyer:** writing – original draft, conceptualization, funding acquisition, methodology, visualization, writing – review and editing, formal analysis, project administration, supervision, resources, data curation, validation.

## Ethics Statement

This study did not involve vertebrate animals or humans. Ethical or IRB approval was not needed. Ethics approval for this research was not required because the experiments were conducted using an unregulated insect.

## Conflicts of Interest

The authors declare no conflicts of interest.

## Supporting information


Supporting File

## Data Availability

The data that support the findings of this study are available in the Supporting Information Material of this article.
